# Supplementation with Lentinan Improves the Colostrum Quality of Holstein Dairy Cows and the Immunity and Antioxidant Capacity of Newborn Calves

**DOI:** 10.3390/ani15060835

**Published:** 2025-03-14

**Authors:** Yinghao Huang, Yapeng Hu, Longfei Lv, Dian Wang, Xiao Li, Sijia Liu, Zhao Zhuo, Caiyun Fan, Jianbo Cheng

**Affiliations:** 1 College of Animal Science and Technology, Anhui Agricultural University, Hefei 230036, China; huangyinghao798@163.com (Y.H.); 18567032653@163.com (Y.H.); lvlondfei3259@163.com (L.L.); 13685693231@163.com (X.L.); liusijia1214@163.com (S.L.); zhuozhao90@163.com (Z.Z.); 2 National Center of Technology Innovation for Dairy, Hohhot 010010, China; wd@yourandairy.com; 3 Inner Mongolia Youran Dairy Group Limited, Hohhot 010010, China

**Keywords:** newborn calves, transfer passive immunity, colostrum, lentinan

## Abstract

The aim of this study was to evaluate the effects of lentinan (LNT) on the quality of colostrum produced by perinatal dairy cows and the health status of their newborn calves. A total of 40 expectant Holstein cows, matched for parity and expected date of delivery, were selected and randomly divided into four groups: a control group fed a total mixed diet (TMR); a low LNT group (10 g/d, LLNT); a medium LNT group (20 g/d, MLNT); and a high LNT group (40 g/d, HLNT). The study commenced 21 days prior to parturition and continued for three weeks. Colostrum was collected from the cows immediately after delivery and subsequently fed to the newborn calves. The results indicated that colostrum milk protein production and IgG production in the MLNT group were significantly increased (*p* < 0.05). Following colostrum gavage, serum SOD and IgG in both the MLNT and HLNT groups showed significant increases (*p* < 0.05), while MDA and IL-1β levels were significantly decreased (*p* < 0.05). Moreover, calves in the MLNT and HLNT groups experienced lower incidences of diarrhea, pneumonia, and overall morbidity compared to those in the control group. In conclusion, LNT enhanced the quality of colostrum in perinatal cows and contributed to the health of newborn calves through colostrum. This study offers new research avenues for improving the health of newborn calves and provides a theoretical foundation for the development of LNT as a novel feed additive.

## Simple Summary

This study demonstrated that LNT improved colostrum composition, increased serum antioxidant enzyme and immunoglobulin levels in newborn calves, and reduced morbidity in calves. These findings offer new insights into the healthcare of newborn calves, suggesting that the inclusion of active substances in the diets of pregnant heifers may yield beneficial effects. Nonetheless, the impact of LNT supplementation on the placenta and fetus during pregnancy remains unclear, indicating a need for further research to elucidate the effects of LNT on the pregnant cow-calf system.

## 1. Introduction

The cow’s placenta inhibits the transfer of macromolecular substances from the mother to the fetus, thereby preventing the transport of immunoglobulins. As a result, calves are born without immunoglobulins in their blood [1]. Consequently, colostrum serves as the sole source of immunoglobulins essential for the survival of newborn calves, which transfer passive immunity (TPI) through the consumption of high-quality colostrum [2]. Increasing evidence underscores the significance of colostrum for calf health. The absorption of immunoglobulins by newborn calves ceases approximately 24 h after birth [3]. Calves that do not receive high-quality colostrum are unable to absorb adequate amounts of IgG, which can lead to increased morbidity and mortality rates. The early stages of colostrum formation and mammary gland development involve the transfer of maternal immunoglobulins from the blood to mammary gland secretions. This process commences 3 to 4 weeks prior to calving and concludes shortly before calving [4]. The mechanisms underlying colostrum production suggest that improving colostrum quality may be achieved by enhancing maternal immunity levels, particularly the concentration of immunoglobulins in the serum of dairy cows during the prenatal period. This, in turn, would provide a greater supply of immunoglobulins to newborn calves, thereby promoting their health, growth, and development during the early stages of life. Numerous studies have explored methods to enhance the quality of colostrum by modifying the nutritional formula provided to dairy cows during the pre-partum period. One study demonstrated that incorporating choline into the diet of transitional dairy cows resulted in increased colostrum production [5]. Similarly, another investigation found that supplementing mannan oligosaccharides also led to an enhancement in colostrum production [6]. Additionally, some researchers have reported that the immune globulin content in the colostrum of dairy cows was elevated through the supplementation of nicotinic acid [7]. Furthermore, a study introduced β-glucan into the diets of transition period (TP) dairy cows, revealing improvements in the immunoglobulin levels of both colostrum and calf serum [8]. Collectively, these studies validate the potential for enhancing colostrum quality in dairy cows through the provision of specific active substances.

Lentinan (LNT) is a macromolecular polysaccharide and a β-glucan characterized by a unique triple helix structure [9]. Currently, LNT is extensively utilized as a nutraceutical to enhance health and aid in disease prevention. It exhibits a range of biological activities, including antioxidant, anticancer, anti-inflammatory, and antiviral properties [10]. The primary techniques for extracting polysaccharides from natural sources encompass hot water extraction, subcritical water extraction, enzymatic hydrolysis, and ultrasonic-assisted extraction. The varying extraction methodologies and subsequent purification processes significantly influence the purity and cost of LNT [9]. LNT is widely recognized for its immunomodulatory properties. As an immune enhancer, LNT stimulates the maturation, differentiation, and proliferation of key immune cells, thereby improving their responsiveness to biologically active factors such as lymphokines and hormones [8]. Recent studies have demonstrated that LNT (98%, Solarbio Science & Technology Co., Ltd., Beijing, China) can enhance the production of interferon gamma (IFN-γ), IgG, and IgM [11,12]. Furthermore, another study demonstrated that LNT (98%, Solarbio Science & Technology Co., Ltd., Beijing, China) effectively reversed dexamethasone-induced immunosuppression in mice and enhanced the levels of IgA by modulating T lymphocytes [13]. Existing research has confirmed the great potential of LNT in regulating the immunity of animals and increasing serum immunoglobulin levels.

Improving colostrum quality and promoting TPI in newborn calves, thereby enhancing early calves’ health, presents a significant challenge in dairy production. Numerous studies have confirmed the critical role of LNT in increasing immunoglobulin levels in the body. However, there is currently a lack of research focused on enhancing colostrum quality in TP dairy cows through the addition of LNT to animal feed, and its subsequent impact on calf health. We hypothesized that LNT could enhance the quality of colostrum from TP cows and, consequently, improve calf health via colostrum consumption. This experiment investigated the effects of LNT on the quality of colostrum from TP cows and its influence on oxidative stress, inflammatory factors, and immunoglobulin levels in calves. We believe that LNT represents a promising nutritional strategy for improving calf TPI, and that this novel feed additive can effectively enhance the health status of newborn calves.

## 2. Materials and Methods

### 2.1. Animal Ethics

This experiment was approved by the Laboratory Animal Management and Animal Ethics Committee of Anhui Agricultural University (No. SYDW-P20190600601).

### 2.2. Animals and Experimental Design

This experiment was conducted in an ecological pasture in Inner Mongolia, China, in 2024. It involved 40 Holstein cows selected based on similar physiological status, parity (2 ± 0.2), and expected date of delivery. A randomized block design was employed in this study. The cows were randomly divided into four groups: (1) a control group (CON, *n* = 10), which was fed a total mixed diet (TMR); (2) a low LNT group (LLNT, *n* = 10), where each cow was fed an additional 10 g/d LNT; (3) a medium LNT group (MLNT, *n* = 10), where each cow received an additional 20 g/d LNT; and (4) a high LNT group (HLNT, *n* = 10), where each cow was supplemented with an additional 40 g/d LNT. The trial commenced 21 days prior to the expected calving date and lasted for three weeks. During this period, the cows were maintained in an open environment where they could move freely. Cows from different treatment groups were housed in separate designated areas within the same barn. Unlimited access to clean water was provided, and a sufficient amount of TMR (110%) was supplied to ensure continuous availability of food and water. Feeding occurred three times daily at 09:00, 14:00, and 20:00 h. The health status of the cows was monitored daily throughout the experiment, and no additional medications were administered during this period. The LNT powder, with a purity greater than 51.99%, was provided by Anhui Fengcao Biotechnology Co., Ltd. (Hefei, China). The TMR design adheres to the standards set by the National Research Council (NRC) for dairy cattle [14]. The composition and nutritional ingredients of the TMR are detailed in Table 1.

After delivery, the calves received care from professional nurses, who administered 4 L of colostrum within the first 30 min. The colostrum provided to each calf was sourced from its mother. Subsequently, the calves were placed on Calf Island. The incidence of diarrhea, pneumonia, and other diseases in the calves was recorded over the next 2 weeks. We recorded the weight of the calves at the beginning and end of the experiment, that is, the weight of the calves at birth and at 2 weeks of age.

### 2.3. Sample Collection

Colostrum samples were collected following the birth of the cows. The samples were then divided into two portions, with one portion mixed with a preservative for the purpose of testing the milk composition. Additionally, one aliquot was frozen at −20 °C.

Blood samples were collected 24 h after the calves were fed colostrum. The samples were obtained from the calves’ jugular veins using vacuum blood collection tubes. Immediately following collection, the serum was separated by centrifugation at 3000 rpm for 20 min at 4 °C. The serum samples were subsequently stored at −40 °C for further analysis.

TMR samples were collected for nutritional analysis. After collection, the samples were placed in an oven at 65 °C for 48 h to remove moisture, followed by crushing using a pulverizer (Model 1188Y, Thomas Wiley, Monmouth, NJ, USA). Crude protein (CP) was determined using a Scio KT260 (FOSS, France Telecom Industries, Copenhagen, Denmark), while neutral detergent fiber (NDF) and acid detergent fiber (ADF) were analyzed using a fiber analyzer (Model 2000i, Ankom, Macedonia, NY, USA). These methods are based on techniques established by Van Soest. The ash content was determined according to AOAC standards, with specific reference to the work of Jiang et al. [15]. The procedures employed adhered to the guidelines established by the NRC [14].

### 2.4. Colostrum Composition Parameters

The colostrum yield was recorded, and the lactose, milk fat, and milk protein contents in the colostrum were analyzed at the Guangming DHI Testing Center (Guangming Dairy Co., Ltd., Shanghai, China). Special kits from Hengyi Biotechnology Co., Ltd. (Yangzhou, China) were utilized to determine the levels of IgA, IgG, and IgM in the colostrum.

### 2.5. Serum Biochemical Parameters

Superoxide dismutase (SOD), catalase (CAT), glutathione peroxidase (GSH-PX), total antioxidant capacity (TAOC), malondialdehyde (MDA), and tumor necrosis factor-alpha (TNF-α), as well as interleukins IL-1β and IL-6, and immunoglobulins IgA, IgG, and IgM, were measured in serum using specialized kits. These kits were provided by Hengyi Biotechnology Co., Ltd. (Yangzhou, China).

### 2.6. Statistical Analysis

All the experimental data were analyzed using SPSS software version 20.0 (IBM Corp., Armonk, NY, USA). Differences among the treatment groups were assessed through ANOVA and Duncan’s multiple range test. The data are expressed as mean ± SEM. A *p*-value of less than 0.05 is considered statistically significant, whereas a *p*-value between 0.05 and 0.10 indicates a statistical trend.

## 3. Results

### 3.1. Colostrum Yield and Composition

The results indicated that, compared to the CON group, the MLNT group exhibited a trend towards increased colostrum milk protein (0.05 < *p* < 0.1, Table 2). Additionally, the MLNT group demonstrated a significant increase in colostrum milk protein production (*p* < 0.05, Table 2). While colostrum lactose production also showed a tendency to increase in the MLNT group (0.05 < *p* < 0.1, Table 2), no significant differences were observed in colostrum lactose and overall colostrum production among the groups.

### 3.2. Colostrum Immunoglobulin Concentration and Production

The experimental results indicated that the concentration of IgG in colostrum was significantly higher in the MLNT and HLNT groups compared to the CON group (*p* < 0.05, Table 3). Additionally, the IgG production in the colostrum of the MLNT group was significantly greater than that observed in the LLNT and control groups (*p* < 0.05, Table 3).

### 3.3. Weight of Calf

**Figure 1:**
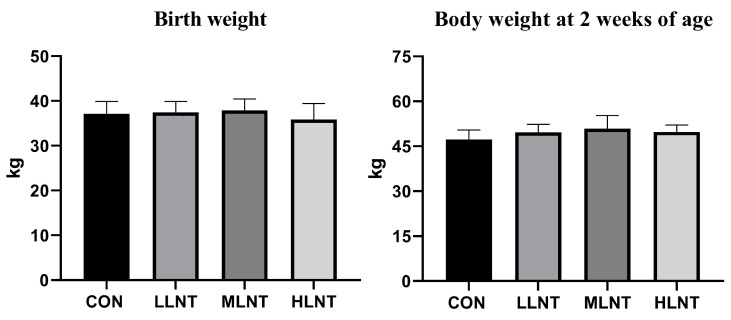
Weight changes of calves in different treatment groups.

The test results indicated no statistically significant difference in calf birth weight between the control group and each treatment group. Furthermore, no significant differences were observed in calf weights among groups after two weeks of life (Figure 1).

### 3.4. Serum Oxidative Stress Parameters in Calves

The results indicated that serum levels of SOD and CAT in the MLNT and HLNT groups were significantly higher than those in the CON group 24 h after colostrum administration (*p* < 0.05, Table 4). Furthermore, serum MDA levels were significantly reduced in the MLNT and HLNT groups (*p* < 0.05, Table 4).

### 3.5. Serum Inflammatory Factors and Immune Globulin Parameters in Calves

The experimental results indicated that serum IL-1β levels were significantly reduced in all dose groups when compared to the CON group (*p* < 0.05, Table 5).

In addition, serum IgG levels were significantly higher in calves in the MLNT and HLNT groups (*p* < 0.05, Table 6).

### 3.6. Incidence of Newborn Calves

**Figure 2:**
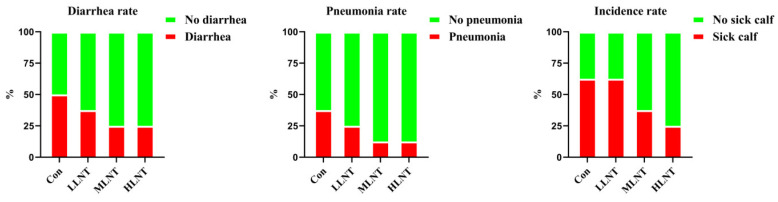
Morbidity of calves in different treatment groups.

The results of this experiment showed that colostrum from cows after LNT feeding significantly reduced the rates of diarrhea and pneumonia in their calving calves. Colostrum from cows in MLNT and HLNT groups reduced calf morbidity (Figure 2).

## 4. Discussion

The initial secretions from the dairy mammary gland are vital for the calf’s early development and health [16]. Colostrum is rich in various bioactive molecules that facilitate immune maturation, intestinal development, and the early microbial colonization of the calf. It contains substantial quantities of immunoglobulins, which are essential components of the immune response, aiding newborn calves in combating bacterial and viral infections [17]. A more extreme manifestation of the new calf’s physiology is that the maximum absorption of immunoglobulins occurs within the first few hours after birth. After approximately 24 h, the intestines lose the capacity to absorb immunoglobulins [18]. It seems that enhancing colostrum quality can be achieved by addressing immunosuppression in TP cows, thereby improving the health of newborn calves through effective colostrum management. LNT is widely recognized for its immunomodulatory properties, and recent studies have demonstrated that LNT can promote immunoglobulin production and alleviate immune suppression [13]. Specifically, lentinan has been shown to enhance the synthesis of cytokines such as IL-2 and IL-4. IL-2 plays a crucial role in driving the growth of T cells and enhancing the activity of natural killer (NK) cells, thereby strengthening the body’s immune response [19]. By promoting the proliferation and differentiation of immune cells, particularly B cells, LNT facilitates the production of immunoglobulins in the animal body. Studies have demonstrated that following oral administration of LNT for seven days, there is a significant increase in the number of lymphocytes in immunosuppressed mice, accompanied by elevated levels of intestinal secretory IgA [20]. Additionally, a study utilizing dexamethasone to establish a mouse immunosuppression model indicates that LNT enhances immunoglobulin levels by modulating T lymphocytes, ultimately reversing the immunosuppressive state and increasing serum antibody levels [13]. Additionally, evidence suggests that LNT can enhance immune cell function, leading to increased immunoglobulin production [21]. LNT modulates immune responses through interactions with various receptors on immune cells. Research has demonstrated that LNT can bind to the pattern recognition receptor dectin-1, thereby activating immune functions [22]. In this experiment, we observed a significant increase in milk protein production in the colostrum of the MLNT group following LNT administration. Additionally, IgG levels in the colostrum of both the MLNT and HLNT groups showed significant increases. Furthermore, the IgG protein content in the MLNT group also increased significantly. These findings indicate that the quality of colostrum in both the MLNT and HLNT groups has been markedly improved, aligning with our initial hypothesis. It appears that LNT may enhance the immunosuppressive state of cows during the early stage of TP, potentially increasing the number or functionality of immune cells, which may lead to a greater quantity of immunoglobulins entering the colostrum.

The transition from the protected environment of the uterus to the external world represents a critical physiological challenge for newborn calves. During this period, the calves encounter multiple survival pressures that can induce oxidative stress. For instance, the shift from the hypoxic conditions within the uterus to the elevated oxygen levels in the external environment results in a rapid increase in reactive oxygen species (ROS). This sudden surge in ROS can overwhelm the immature redox system of the calves, triggering pronounced oxidative stress [23]. In mammals, the neonatal antioxidant system is not fully developed at birth, contributing to the significant oxidative stress observed in newborn calves. This deficiency in antioxidant capacity typically improves over time, leading to a restoration of REDOX balance by approximately seven days post-birth [24]. At the same time, oxidative stress during this period further interferes with the normal function of immune cells in calves, which leads to a decline in immunity in calves [25]. Current research indicates that enhancing the health of newborn calves can be achieved by optimizing the health of the maternal body during pregnancy through the supplementation of antioxidants, trace elements, and other bioactive nutrients. Some studies have administered a variety of organic trace mineral supplements to pregnant cows. A study investigating organic trace mineral supplementation in heifers during late gestation found that the incidence of respiratory and digestive diseases in treated calves was significantly lower than that in control calves. Furthermore, the treatment group exhibited a significant increase in the number of immune cells, resulting in a notable enhancement of immunity [26]. Several studies have investigated the prenatal supplementation of ruminal methionine in cows. The results indicated that neonatal calves in the treatment groups exhibited reduced levels of inflammatory proteins and improved immune cell functions, including cell adhesion, chemotaxis, oxidative stress response, and Toll-like receptor signaling, when compared to the control groups [27]. Similar results have been found in other mammalian studies, and one study, in which pregnant sows were fed with herbal antioxidants, showed improved survival of newborn piglets in treatment groups, increased serum antioxidant enzyme levels, and decreased levels of oxidative stress standards [28]. For dairy cows in the transition period, inflammation is often associated with oxidative stress. The free radicals generated from the mobilization of adipose tissue are a significant contributor to this inflammatory response. Additionally, research has demonstrated that the pathways involved in inflammation and oxidative stress are interconnected [29]. In this experiment, we observed a similar phenomenon, where serum levels of SOD and CAT were significantly elevated, while MDA levels were significantly reduced in the newborn calves of the MLNT and HLNT groups. This phenomenon may be attributed to the role of LNT in alleviating oxidative stress and enhancing the immune function of TP cows. Substantial evidence supports the notion that maternal health during pregnancy plays a critical role in determining neonatal health [30,31]. In addition, inflammatory factors can also be transferred from the mother to the fetus to regulate immune and inflammatory responses [32,33].

The results of this experiment indicated that serum IL-1β levels in calves from the MLNT and HLNT groups were significantly decreased, while IgG levels were significantly increased. Additionally, the incidence of disease in the MLNT and HLNT groups was lower than that observed in the CON group. These findings suggest that the TPI in newborn calves from the MLNT and HLNT groups was superior to that in the CON group. This phenomenon appears to be attributable to the high-quality colostrum produced by the TP cows following LNT supplementation. Evidence supports the feasibility of administering maternal antioxidants or immunomodulators to enhance colostrum quality and neonatal health. Studies involving the addition of Forsythia suspensa extract (FSE) to the diets of pregnant sows demonstrated that FSE increased the levels of colostrum milk fat, milk protein, and IgM, thereby improving the immunity of piglets [34]. Additionally, another study found that incorporating resveratrol into the diets of sows improved offspring intestinal morphology and reduced intestinal inflammation and diarrhea [35]. Colostrum serves as the primary source of energy and nutrients for the calf during its initial hours of life [36]. It is abundant in immunoglobulins, which confer immunity to the calf in the first weeks post-birth. Additionally, colostrum is crucial for safeguarding the calf’s gastrointestinal tract from pathogenic organisms [37]. Accordingly, Infascelli et al. reported that supplementing the diet of transition buffalo cows with Aloe arborescens extract improves passive immune transfer in newborn calves by increasing colostrum IgG content. The authors attributed the results to a stimulation of quiescence macrophages and other immune cells by the action of surface polysaccharides of cell Aloe on the PPR (patters recognized receptors) [38]. The adequacy of colostrum feeding is crucial in determining whether a calf achieves successful passive immunization (SPI) or experiences failed passive immunization (FPI) [39]. Research indicates a strong correlation between the failure of passive immunization and increased calf mortality and morbidity [40]. Furthermore, calves that suffer from FPI are at a heightened risk of infections caused by rotavirus and Cryptosporidium, as well as an overall increased incidence of diarrhea [41]. The etiology of diarrhea and pneumonia in calves is multifaceted, encompassing pathogen infection, environmental changes, and malnutrition. In this study, it is hypothesized that environmental pathogens may compromise calves with weakened passive immunity, particularly those in the CON group [42,43]. Conversely, calves in the treatment groups, notably the MLNT and HLNT groups, exhibited enhanced immune responses against external pathogens, which may be attributed to superior colostrum quality. It has been demonstrated that LNT can effectively reduce the levels of inflammatory factors in the blood of lactating cows [19]. Furthermore, LNT supplementation has been shown to increase the percentage of lymphocytes in rabbits [44]. Additionally, LNT supplementation in mice enhances the expression of MHC II, CD80/CD86, and Toll-like receptors (TLR2/TLR4), regulates T-cell populations, and increases IL-10 secretion. These findings suggest the potential preventive role of LNT in malaria treatment [45]. In this experiment, the overall morbidity of calves in the MLNT and HLNT groups was lower than that observed in the CON group, indicating that these calves benefited from more effective passive immunization. For calves, this enhanced colostrum intake reduces the incidence of diarrhea and pneumonia, thereby improving the overall health of newborn calves.

## 5. Conclusions

In conclusion, maternal supplementation with LNT in dairy cattle during late gestation demonstrated dual benefits: enhanced colostrum quality through increased milk protein synthesis and elevated IgG concentrations. Improved neonatal health outcomes were achieved through colostrum-mediated effects, including upregulated antioxidant capacity (SOD, CAT), enhanced immune parameters (IgG), and reduced oxidative stress (MDA) and inflammatory markers (IL-1β). Notably, there was no significant difference in the direct effects observed between the MLNT and HLNT groups, indicating that a dosage of 20 g of LNT per cow per day is sufficient to achieve its intended efficacy. This experiment offers a novel approach to improving calf health and establishes a theoretical foundation for the application and promotion of LNT as a new feed additive.

## Figures and Tables

**Table 1:** TMR composition and nutritional levels (DM).

Items	Content (%)
Whole corn silage	36
DDGS ^1^	7
Corn meal	8
Soybean meal	11
Flaked corn	10
Cottonseed	4
Oat hay	22
Prenatal Premix ^2^	2
Total	100
Nutrient levels ^3^	
CP	12.66
EE	2.88
NDF	38.41
ADF	25.91
Ash	7.86
Calcium	0.52
Phosphorus	0.45
NE_L_ ^4^, Mcal/kg DM	1.40

^1^ DDGS, distiller dried grains with soluble. ^2^ One kg prenatal premix contained the following: VA 200, 000 IU, VD 60, 000 IU, Fe 400 mg, Cu 450 mg, Zn 2, 000 mg, Mn 550 mg, Se 15 mg, Co 20 mg. ^3^ CP, crude protein; EE, ether extract; NDF, neutral detergent fiber; ADF, acid detergent fiber. ^4^ Predicted values from NRC (2021) model [14].

**Table 2:** Effects of different doses of LNT on colostrum in transitional cows.

Items	Treatment				SEM ^1^	*p*-Value
	Con	LLNT	MLNT	HLNT		
Milk fat, %	6.59	6.48	6.58	6.90	0.30	0.969
Milk protein, %	16.22	17.79	19.03	18.91	0.42	0.060
Lactose, %	3.05	3.07	3.17	3.12	0.10	0.979
Total solids, %	26.19	27.73	28.61	29.26	0.60	0.315
Milk fat yield, kg	0.57	0.59	0.74	0.69	0.04	0.490
Milk protein yield, kg	1.41 ^b^	1.53 ^b^	2.22 ^a^	1.83 ^ab^	0.10	0.014
Lactose yield, kg	0.26	0.26	0.36	0.30	0.01	0.074
Milk yield, kg/d	8.67	8.79	11.56	9.73	0.47	0.101
4% FMT, kg/d	8.58	8.94	11.09	10.33	0.64	0.488

^1^ SEM = Standard error of means; ^a,b^ Means within a row with different superscripts differ (*p* < 0.05).

**Table 3:** Effects of different doses of LNT on colostrum immunoglobulin in transitional cows.

Items	Treatment				SEM ^1^	*p*-Value
	Con	LLNT	MLNT	HLNT		
IgA, g/L	27.15	25.66	24.40	23.86	0.82	0.513
IgG, g/L	34.45 ^b^	37.46 ^ab^	43.59 ^a^	45.49 ^a^	2.58	0.037
IgM, g/L	10.93	10.37	9.69	9.82	0.62	0.902
IgA yield, g	234.83	227.80	281.68	235.73	14.82	0.576
IgG yield, g	290.63 ^c^	330.21 ^bc^	502.49 ^a^	434.85 ^ab^	24.23	0.003
IgM yield, g	93.67	90.94	111.61	96.04	7.76	0.801

^1^ SEM = Standard error of means; ^a,b,c^ Means within a row with different superscripts differ (*p* < 0.05).

**Table 4:** Effects of colostrum from cows supplemented with different doses of LNT on oxidative stress in their calves.

Items	Treatment				SEM ^1^	*p*-Value
	Con	LLNT	MLNT	HLNT		
SOD, U/mL	115.21 ^b^	124.53 ^ab^	135.26 ^a^	135.73 ^a^	2.69	0.012
CAT, U/lmL	33.89 ^b^	38.75 ^b^	63.47 ^a^	46.11 ^b^	3.31	0.004
GSH-PX, U	488.29	512.51	460.16	485.94	11.79	0.500
TAOC, mM/L	1.17	1.14	1.14	1.14	0.01	0.237
MDA, umol/L	20.00 ^a^	15.87 ^ab^	14.61 ^b^	13.96 ^b^	0.81	0.003

^1^ SEM = Standard error of means; ^a,b^ Means within a row with different superscripts differ (*p* < 0.05).

**Table 5:** Effect of colostrum from cows supplemented with different doses of LNT on serum inflammatory factors in their calves.

Items	Treatment				SEM ^1^	*p*-Value
	Con	LLNT	MLNT	HLNT		
TNF-α, ng/mL	0.78	0.85	0.83	0.62	0.04	0.138
IL-1β, ng/mL	3.82 ^a^	2.85 ^b^	2.46 ^b^	2.06 ^b^	0.19	0.003
IL-6, ng/mL	2.76	4.88	3.94	2.15	0.47	0.170

^1^ SEM = Standard error of means; ^a,b^ Means within a row with different superscripts differ (*p* < 0.05).

**Table 6:** Effect of colostrum from cows supplemented with different doses of LNT on serum immunoglobulins in their calves.

Items	Treatment				SEM ^1^	*p*-Value
	Con	LLNT	MLNT	HLNT		
IgA, g/L	1.09	1.08	1.03	1.05	0.02	0.737
IgG, g/L	15.51 ^b^	18.52 ^ab^	22.96 ^a^	21.16 ^a^	0.98	0.033
IgM, g/L	2.26	2.26	2.40	2.88	0.18	0.591

^1^ SEM = Standard error of means; ^a,b^ Means within a row with different superscripts differ (*p* < 0.05).

## Data Availability

The original contributions presented in this study are included in the article. Further inquiries can be directed to the corresponding authors.
